# Animal Models for COVID-19: Hamsters, Mouse, Ferret, Mink, Tree Shrew, and Non-human Primates

**DOI:** 10.3389/fmicb.2021.626553

**Published:** 2021-08-31

**Authors:** Shuyu Shou, Menghui Liu, Yang Yang, Ning Kang, Yingying Song, Dan Tan, Nannan Liu, Feifei Wang, Jing Liu, Youhua Xie

**Affiliations:** ^1^Key Laboratory of Medical Molecular Virology, School of Basic Medical Sciences, Fudan University, Shanghai, China; ^2^Children’s Hospital, Fudan University, Shanghai, China

**Keywords:** COVID-19, SARS-CoV-2, ACE2 (angiotensin converting enzyme 2), animal model, pathogenesis

## Abstract

Severe acute respiratory syndrome coronavirus 2 (SARS-CoV-2) is a novel coronavirus causing acute respiratory tract infection in humans. The virus has the characteristics of rapid transmission, long incubation period and strong pathogenicity, and has spread all over the world. Therefore, it is of great significance to select appropriate animal models for antiviral drug development and therapeutic effect evaluation. Here, we review and compare the current animal models of SARS-CoV-2.

## Introduction

In early December of 2019, a new coronavirus disease (coronavirus disease 2019, COVID-19) caused by severe acute respiratory syndrome coronavirus 2 (SARS-CoV-2) was reported in Wuhan, China (Li et al., 2020). SARS coronavirus (SARS-CoV), Middle East respiratory syndrome coronavirus (MERS-CoV), and SARS-CoV-2 all belong to the *Betacoronavirus* genus of *Coronaviridae* family and have typical coronavirus genome structure (Zhu et al., 2020). Bats are suspected to be the natural host of SARS-CoV-2 (Banerjee et al., 2019), while other animals such as turtles, pangolins, snakes, cats, rabbits, ferrets, and monkeys have been suggested as potential intermediate hosts (Liu et al., 2020a; Wu et al., 2020). The spike (S) protein on the surface of SARS-CoV-2 is responsible for binding angiotensin converting enzyme 2 (ACE2) receptor on target cell surface (Guo et al., 2020). The interaction between viruses and host-specific receptors is an important factor that limits species tropism of the pathogen (Douam et al., 2015).

Severe acute respiratory syndrome coronavirus 2 infection may cause mild to severe symptoms. Severe outcomes include acute respiratory distress syndrome (ARDS), sepsis, septic shock, multi-organ failure, and death (Harapan et al., 2020). According to one survey, 80.9% infections were mild, 4.7% eventually developed critical symptoms, and the overall case fatality rate was 2.3% (Zhang, 2020) which is lower than MERS (34.4%) and SARS (9.5%) (Munster et al., 2020b). However, some patients (especially elderly patients with diabetes or coronary heart disease) can deteriorate in a short period of time and rapidly progress to multi-organ failure or death (Chen N. et al., 2020; Zhou F. et al., 2020). Other conditions associated with high mortality in adults include hypertension and other preexisting respiratory diseases (Williamson et al., 2020). Similar to SARS and MERS, fast deterioration in COVID-19 patients has been linked to the development of cytokine storm (Huang et al., 2005; Zhou et al., 2014). Serum levels of pro-inflammatory cytokines, such as interleukin-6 (IL-6), IL-1β, IL-2, IL-8, IL-17, granulocyte-colony stimulating factor (G-CSF), monocyte chemotactic protein-1 (MCP-1), interferon-γ-inducible protein-10 (IP-10), and interferon (IFN), are upregulated in most patients with severe symptoms (Huang et al., 2020; Tan et al., 2020) as a result of excessive immune response to SARS-Cov-2 infection (Hu et al., 2020). Besides, it has been observed that most severe cases have lymphopenia, displaying a sharp decrease in the number of CD4^+^ T cells, CD8^+^ T cells, B cells, and natural killer cells. Total leukocyte number was not remarkably changed in such patients, but neutrophils were increased (Huang et al., 2020; Tan et al., 2020), and the increase in neutrophil-to-lymphocyte ratio (NLR) has been associated with disease severity and poor clinical outcome (Cao, 2020). Such anomalies in cell counts and NLR will eventually disappear in convalescent patients (Zheng et al., 2020).

Animal models are valuable tools for elucidating the pathogenesis of SARS-CoV-2 and evaluating antiviral therapeutics and vaccines. We searched the literature for currently used COVID-19 animal models. This review attempts to summarize progresses made since the discovery of SARS-CoV-2 in the development and application of relevant animal models, and discusses each model’s strength and weakness (Table 2). Details about each animal model are listed in Table 1, including gender, age, inoculation, replication, pathology, immune response, and clinical signs.

**TABLE 1:** Experimental animal models for SARS-CoV-2.

Animal	Gender	Age	Inoculation		Replication	Immune response	Clinical signs	Pathology	Lethality	Animal-animal trans mission	References
			Dosage	Route							
Tree shrew	Male and female	Adult (1 year), aged (5–6 years)	10^7^ TCID_50_	IN, OR, OC	RNA in lung conjunctive	Increase in leukocyte, lymphocyte, granulocyte; no IgM and neutralizing antibodies detected	NR	Lung radiographic abnormalities; pulmonary punctate hemorrhage	Non-lethal	NR	Xu L. et al., 2020
Tree shrew	Male and female	Young (6–12 months), adult (2–4 years), old (5–7 years)	10^6^ PFU	IN	Lung, digestive tissues, heart, pancreas, kidney	NR	Increased body temperature	Mild pulmonary abnormality; mild changes in some extrapulmonary organs	Non-lethal	NR	Zhao et al., 2020
Syrian hamster	Male and female	6–10 weeks	10^5^ PFU	IN	Mainly in nasal turbinate, trachea, lung tissues, and intestine	Neutralizing antibody response, IFN-γ, IL-4, IL-6, TNF-α, and IL-12p40 upregulated	Weight loss, lethargy, ruffled fur, hunched back posture, rapid breathing	Pneumonia, diffuse alveolar damage with extensive apoptosis in lung; mild changes in heart and spleen	Non-lethal	Yes	Chan et al., 2020
Syrian hamster	Male	4–5 weeks	8 × 10^4^ TCID_50_	IN	Lung, nasal turbinate, kidney, duodenum	Neutralizing antibody response	Weight loss	Pneumonia, mononuclear cell infiltrate; nasal epithelial attenuation	Non-lethal	Yes	Sia et al., 2020
Syrian hamster	Female	Yound (1 month), adult (7–8 months)	10^5^.^6^ or 10^3^ PFU	IN, OC	Trachea, lung, nasal turbinate, brain, or olfactory bulb	Neutralizing antibody response	Weight loss	Severe pneumonia; lung radiographic abnormalities	Non-lethal	Yes	Imai et al., 2020
Syrian hamster	Male and female	10–12 weeks	5 × 10^4^ or 5 × 10^5^ TCID_50_	IN	Lung, nares, trachea, heart, gastrointestinal tract, brain, spleen, liver, kidney	Macrophages and CD3^+^ T lymphocytes infiltration in lung	Weight loss, respiratory distress	Moderate to severe interstitial pneumonia	Lethal	NR	Tostanoski et al., 2020
*RAG2* KO Syrian hamster	Male and female	11–12 weeks	10^4^ PFU	IN	Lung, heart, liver, spleen, intestine, brain, kidney	NR	Weight loss	Severe pneumonia	Lethal	NR	Brocato et al., 2020
Roborovski dwarf hamster	Male and female	5–7 weeks	10^5^ or 5,000 PFU	IN	RNA in bucco-laryngeal swabs, serum sample; lung, jejunal, kidney	NR	Decrease of body temperatures, weight loss, snuffling, dyspnea, and ruffled fur	Severe fulminant pneumonia	Lethal	NR	Trimpert et al., 2020
Chinese hamster	Male and female	5–7 weeks	10^5^ PFU	IN	Lung, bucco-laryngeal swabs, blood samples	NR	Weight loss, subtle drops in body temperatures	Bronchitis, pneumonia	Non-lethal	NR	Bertzbach et al., 2020
BALB/c mice	Female	Aged (9 months), young (6 weeks)	1.6 × 10^4^ PFU MASCp6	IN	Lung, trachea, heart, liver, spleen, brain, feces	IL-1β, IL-6, and IL-5 upregulated	NR	Mild to moderate interstitial pneumonia	Non-lethal	NR	Gu et al., 2020
BALB/c or C57BL/6J mice	Female	Young (4–6 weeks), adult (8–9 weeks)	10^4^.^4^ PFU HRB26M	IN	Respiratory tract	NR	Transient weight loss	Mild to moderate pneumonia	Non-lethal	NR	Wang et al., 2020
BALB/c mice	Female	Adult (12 weeks), aged (1 year)	10^5^ PFU SARS-CoV-2 MA	IN	Upper and lower airways	IL-3, TNF-α, and IL-1β upregulated in lung	No overt clinical signs	Impaired lung function; mild-to-moderate pneumonia; peribronchiolar lymphocytic inflammation	Non-lethal	NR	Dinnon et al., 2020
BALB/cAnNHsd mice	Female	Young (10 weeks), old (1 year)	10^2^–10^5^ PFU SARS-CoV-2 MA10	IN	Upper and lower airways	IL-6, IL-1α, IL-1β, TNF-α, MCP-1, and IFN-γ upregulated	Weight loss	Severe pneumonia, edema, and diffuse alveolar damage	Lethal	NR	Leist et al., 2020
C57BL/6J mice	Female	10 weeks	10^4^ PFU SARS-CoV-2 MA10	IN	Respiratory tract	NR	Weight loss	Pneumonia	Non-lethal	NR	Leist et al., 2020
BALB/c mice	Female	4–6 weeks	10^5^ PFU SARS-CoV-2 WBP-1	IN	Lung, trachea, turbinate	IL-1β, TNF-α, MCP-1, and IL-10 upregulated in lung	Weight loss	Severe interstitial pneumonia	Lethal	NR	Huang et al., 2021
hACE2 transgenic mice	Male and female	6–11 months	10^5^ TCID_50_ SARS-CoV-2 HB-01	IN	Lung tissues, RNA in intestine	Specific IgG antibodies against the S protein of SARS-CoV-2 at 21 dpi	Slight bristled fur, weight loss	Moderate interstitial pneumonia	Non-lethal	NR	Bao et al., 2020
hACE2 mice	Female	Young (4.5 weeks), aged (30 weeks)	4 × 10^5^ PFU	IN	Respiratory tracts, brain	G-CSF, IFN-γ, IL-9, and MIP-1β upregulated in aged mice	Weight loss in aged mice	Interstitial pneumonia	Non-lethal	NR	Sun S.H. et al., 2020
K18-hACE2 mice	Male and female	8–9 weeks	2.5 × 10^4^ PFU 2019-n-CoV/USA_WA1/2020	IN	Lung, heart, spleen, kidney, brain	IFN-β, IL-6, IL-11, CXCL10, etc., upregulated in lung, lymphopenia	Weight loss, exercise tolerance reduced, pulmonary function decreased	Severe interstitial pneumonia	Lethal	NR	Winkler et al., 2020
K18-hACE2 mice	Male	8–10 weeks	S1SP (400 μg/kg)	IT	None	IFN-γ, IL-6, MCP-1, TNF-α, and IP-10 upregulated	Weight loss	Alveolar inflammation and edema	Non-lethal	NR	Colunga Biancatelli et al., 2021
HFH4-hACE2 mice	Male and female	8–10 weeks	3 × 10^4^ TCID_50_	IN	Lung, eye, heart, brain	Neutralization antibodies, lymphopenia	Weight loss, dyspnea, neurological symptoms	Severe interstitial pneumonia	Lethal	NR	Jiang et al., 2020
hACE2-KI mice	Male and female	8–10 weeks	4 × 10^5^ PFU	IT	NR	TNF-α, IL-1α, IL-1β, and MCP-1 upregulated	NR	Severe pneumonia, ARDS	Non-lethal	NR	Hong et al., 2021
AdV-hACE2 mice	Male and female	8–10 weeks	10^5^ FFU 2019-n-CoV/USA_WA1/2020	IN, IV/IN	Lung, heart, liver, spleen, and brain	NR	Weight loss	Pneumonia	Non-lethal	NR	Hassan et al., 2020
AdV-hACE2 mice	Male and female	6–8 weeks	10^5^ PFU 2019-nCoV/USA-WA1/2020, SARS-CoV-2/human/CHN/IQTC01/2020	IN	Lung	Virus-specific CD4^+^ and CD8^+^ T cell responses peaked at 8 dpi, TNF, IFN-γ, IL-10, IL-15, IL-6, CCL2, CXCL10, and PDGFb upregulated in lung	Ruffled fur, hunching, difficulty breathing, weight loss	Vascular congestion and hemorrhage; alveolar edema; interstitial inflammatory cell infiltrates	Non-lethal	NR	Sun J. et al., 2020
VEEV-VRP-hACE2 mice	Female	6–8 weeks	10^5^ PFU	IN	Lung	NLR increased	No obvious symptoms	Interstitial pneumonia	Non-lethal	NR	Zhang Y.N. et al., 2020
Ferret	Female	3–4 months	10^5^ PFU	IN	Nasal turbinate, soft palate, tonsils	NR	Fever, loss of appetite	Mild peribronchitis, pneumonia	Non-lethal	NR	Shi et al., 2020
Ferret	Male and female	1–2 years	10^5^.^5^ TCID_50_	IN	Nasal turbinate, trachea, lung, and intestine sections	NR	Increased body temperature, weight loss, occasional coughs	Acute bronchiolitis	Non-lethal	Yes	Kim et al., 2020
Ferret	Female	7 months	5 × 10^2^, 5 × 10^4^, or 5 × 10^6^ PFU	IN	Upper respiratory tract	Neutralizing response	Reduced activity, ruffled fur	Mild bronchiolitis	Non-lethal	NR	Ryan et al., 2021
Mink	Male and female	13 months	5 × 10^6^ PFU	IN	Ear, rectal, and nasal washes; RNA in the nasal turbinates, soft palates, ileum tonsils, all lung lobes, and submaxillary lymph nodes	NR	Weight loss	Severe interstitial pneumonia; severe perivasculitis and moderate peribronchiolitis; nasal mucosa damage of the olfactory region	Non-lethal	Yes	Shuai et al., 2020
Rhesus macaque	Male and female	6–11 years	7 × 10^6^ TCID_50_	IT	Lung, trachea, and bronchus tract; RNA in oropharyngeal, nasal, and rectal swab	Leukocytes and neutrophils decreased, lymphocytes increased	Reduced appetite, weight loss	Lung radiographic abnormalities; interstitial pneumonia	Non-lethal	NR	Shan et al., 2020
Rhesus macaque	Male and female	Adult	2.6 × 10^6^ TCID_50_	IN, IT, OC, OR	Nose, throat, rectal swabs; respiratory tract, lymphoid, and gastrointestinal tissues	Neutralizing response on 10 dpi; IL-1ra, IL-6, IL-10, IL-15, MCP-1, MIP-1β increased on 1 dpi, TGF-α decreased on 3 dpi	Reduced appetite, hunched posture, weight loss, pale appearance, and dehydration	Lung radiographic abnormalities; moderate interstitial pneumonia centered on terminal bronchioles	Non-lethal	NR	Munster et al., 2020a
Rhesus macaque	Male and female	Young (3–5 years), aged (15 years)	10^6^ TCID_50_	IT	Nasal, throat and anal swabs	CD8^+^ and CD4^+^ T cells decline in young animals; lymphocytes decreased	Asthenia, weight loss	Typical interstitial pneumonia	Non-lethal	NR	Yu et al., 2020
African green monkey	Male and female	Adult	4.6 × 10^5^ PFU	IT and IN	Respiratory tract, lymphoid tissue, heart, digestive tract, brain, eyes, urogenital tract	Neutralizing response	Reduced appetite, elevated body temperature	Broncho-interstitial pneumonia	Non-lethal	NR	Woolsey et al., 2021
African green monkey	Male and female	About 16 years	3.61 × 10^6^ PFU	OR, IN, IT, IV, and OC	Viral RNA in buccal, nasal, pharyngeal, bronchial brush, and rectal swabs	IFN-γ, IL-6, IL-4/IL-13, IL-8, IL-1β, and TNF-α upregulated; antibody response	Reduced appetite, respiratory distress	Interstitial pneumonia, ARDS	Lethal	NR	Blair et al., 2021
Cynomolgus macaque	Male and female	Adult	4.75 × 10^6^ PFU	IN, IT, OC	RNA in nasal swabs, throat swabs, anal swabs, feces, blood; lung, trachea, bronchus, and spleen	Virus-specific antibodies; IL-10, IL-1α, IL-8, IL-15, and MCP-1 upregulated	Elevated body temperature, weight loss	Lung radiographic abnormalities; diffuse interstitial pneumonia; inflammation in liver and heart; hyperplasia of mesenteric lymph nodes; mild infiltration of local inflammatory cells in kidney	Non-lethal	NR	Lu et al., 2020
Cynomolgus macaque	Female	Young adult (4–5 years), aged adult (15–20 years)	10^6^ TCID_50_	IT, IN	RNA in nasal, throat, and rectal swabs; respiratory tract, ileum and tracheo-bronchial lymph nodes	Virus-specific antibodies	Serous nasal discharge in one aged animal	Pneumonia, diffuse alveolar damage, pulmonary edema	Non-lethal	NR	Rockx et al., 2020
Common marmoset	Male and female	Adult	10^6^ PFU	IN	RNA in nasal, throat, and anal swabs, blood	Undetectable virus-specific antibodies in serum	Slightly elevated body temperature	Slight infiltration of inflammatory cells into the broken pulmonary septum; swollen hepatocytes; light hemorrhage observed in spleen	Non-lethal	NR	Lu et al., 2020

*IN, intranasal; IT, intratracheal; IG, intragastric; OC, ocular; OR, oral; IV, intravenous; TCID_50_, 50% median tissue culture infectious dose; PFU, plaque forming units; FFU, focus forming unit; dpi, day post infection; NR, not reported; wk, week; mo, month; yr, year; IgM, immunoglobulin M; IgG, immunoglobulin G; IL, interleukin; TNF, tumor necrosis factor.*

**TABLE 2:** Advantages and disadvantages of SARS-CoV-2 animal models.

Animal	Advantages	Disadvantages
Chinese tree shrew	Small size; low feeding cost; short reproductive cycle; evolutionarily close to primates.	No purebreds; limited virus replication and shedding; mild lung infection.
Syrian hamster	Susceptibility to virus; lung damage similar to humans; viral transmission between individuals; the high challenge dose result in death.	Severe disease without typical manifestations of ARDS; short-lived infection kinetics.
Roborovski dwarf hamster	Highly sensitive; rapid and consistent development of clinical signs; lethal outcome; less prone to aggressive behavior.	Not widely used; lack of relevant research tools.
Chinese hamster	Pronounced clinical symptoms; small size; well-characterized genome.	Prolonged pneumonia increasing time and cost.
Wild type mice (mouse-adapted virus strains)	Fast production at low-cost; age-dependent increase in disease severity; mild to lethal disease.	Insusceptible to wild type virus infection; virus replication limited to respiratory system.
hACE2 transgenic mice	Susceptibility to wild type virus; mild to severe lung pathological changes.	Long production time and high cost; suboptimal SARS-CoV-2 replication.
AdV-hACE2 mice	Fast production at low-cost.	hACE2 expressed non-systematically; mild bronchial inflammation associated with AdV; no extrapulmonary manifestations.
Ferret	Display symptoms of cough and fever, and transmission between animals.	Virus replication only in the upper respiratory tract; mild lung pathological damage.
Mink	Severe to fatal infections; lung lesions highly similar to those in human patients.	Not widely used; aggressive animal.
African green monkey	Robust viral replication; lethal outcome in aged; manifestations of ARDS.	High cost; limited animal sources.
Rhesus macaque	Phylogenetically close to human; symptoms similar to human infections; age-dependent increase in disease severity.	Non-lethal outcome; high cost.
Cynomolgus macaques	Phylogenetically close to human; asymptomatic infection in aged animals.	Non-lethal outcome; high cost.
Common marmoset	Phylogenetically close to human; smallest monkey allowing easy handling; breed well in captivity.	Relatively resistant to SARS-CoV-2 infection.

## Chinese Tree Shrew

Chinese tree shrew (*Tupaia belangeri chinensis*), a squirrel-like and rat-sized mammal, is genetically close to primates (Fan et al., 2013). As emerging experimental animal, tree shrews have the advantages of small size, low feeding cost, and short reproductive cycle, and have been used as effective experimental animals to replace primates in biomedical research and drug safety testing, especially for research targeting hepatitis C and B viruses (Yan et al., 1996; Feng et al., 2017).

Two teams have conducted SARS-CoV-2 infection of tree shrews. In one study, after nasal inoculation, tree shrews displayed no clinical symptom except for an increase in body temperature, and SARS-CoV-2 RNA became detectable in nasal, throat, and anal swabs, and one serum sample. Highest virus shedding (copy number 10^5.92^/ml) was recorded with a young tree shrew’s nasal swab at 6 days post inoculation (dpi) and the highest viral load is 10^9.08^/ml obtained using pancreas of one adult tree shrew dissected at 14 dpi (Zhao et al., 2020). In contrast, Xu L. et al. (2020) failed to detect SARS-CoV-2 RNA in throat and anal swabs. In addition, lung was the main site for viral replication, followed by the digestive tissues and heart (Table 1; Zhao et al., 2020). Sections of lung tissues showed local mild lesions, including thickened alveolar septa and interstitial hemorrhage, in both studies. Zhao et al. (2020) also found some scant histopathological changes in brain, heart, liver, trachea, pancreas, etc. (Table 1). Moreover, laboratory examination found that serum albumin decreased after infection, while blood urea nitrogen and alanine aminotransferase increased, similar to the impaired liver and renal function observed during COVID-19 progression (Ronco et al., 2020; Xu L. et al., 2020; Zhang C. et al., 2020). It appears that SARS-CoV-2 only achieves limited virus replication and shedding, as well as insignificant pathogenesis, in tree shrews.

## Hamster

Hamsters are widely used in the research of respiratory viruses (Schaecher et al., 2008; Iwatsuki-Horimoto et al., 2018). Hamster ACE2 binds tightly to the SARS-CoV-2 S protein and mediates its entry (Liu et al., 2021). Therefore, hamsters are a potential infection model for studying the pathogenesis of SARS-CoV-2 infections.

### Syrian Hamsters (*Mesocricetus auratus*)

Syrian hamsters started developing rapid breathing, lethargy, ruffled fur, and weight loss at 2 dpi of SARS-CoV-2. The mean viral loads in the nasal turbinate, trachea, and lung were consistently the highest among all tested tissues (Table 1; Chan et al., 2020). Sia et al. (2020) detected SARS-CoV-2 replication in the duodenum but observed no obvious structural damage or inflammatory infiltration, which is similar to virus replication in the epithelial cells of the terminal ileum and colon of COVID-19 patients.

After infection, Syrian hamsters showed similar pathological manifestations as human COVID-19 pneumonia. Lung tissue displayed focal diffuse alveolar destruction, hyaline membrane formation, and mononuclear cell infiltration (Table 1; Chan et al., 2020). In addition, neutrophil extracellular traps, an important pathogenetic trigger in severe COVID-19 patients (Middleton et al., 2020), was observed in lungs of infected Syrian hamsters (Becker et al., 2021). Computed tomography (CT) detected patchy ground glass opacity with regions of lung consolidation at 2 dpi, and the most severe lung changes occurred at 7–8 dpi (Imai et al., 2020).

Severe acute respiratory syndrome coronavirus 2 infection could cause different degrees of multiple organs lesions in Syrian hamster, including spleen, lymph nodes, kidney, adrenal gland, ovary, etc. Among them, the adrenal gland showed focal to multifocal inflammation (Song et al., 2020). Splenic atrophy and myocardial degeneration could also be observed (Chan et al., 2020), which may be related to the occasional reports of heart failure and lymphopenia in human patients (Chen N. et al., 2020; Huang et al., 2020).

A study found that high-dose intranasal inoculation of SARS-CoV-2 (5 × 10^5^ TCID_50_) in hamsters resulted in severe infection, whose primary manifestation was high levels of virus replication, severe extensive pneumonia, and partial mortality (Tostanoski et al., 2020). The high-dose hamster model recapitulates severe COVID-19 and might help to study the pathogenesis of serious clinical disease and to test antiviral agents. Disrupting of adaptive immunity is also a strategy for developing severe disease models. Cyclophosphamide-treated and *RAG2* knockout (KO) hamsters infected with low doses of virus developed more severe and prolonged disease (Brocato et al., 2020). Low-dose virus-infected *RAG2* KO hamster, which was marked by hemorrhage and severe edema in lung and rapid onset of disease (by 3 dpi), is a uniformly lethal model (Table 1). It demonstrates that the absence of functional B cells and/or T cells exacerbates pathogenesis at an early stage of infection.

The gene expression of the cytokine/chemokine profiles in the lungs of infected hamsters showed a time-dependent trend. IFN-γ and pro-inflammatory cytokines, IL-4, IL-6, TNF-α, and IL-12p40, were induced at 2 dpi and reached peak levels at 4 dpi (Chan et al., 2020). The lymphoid necrosis observed in hamster’s spleen might be associated with its innate immune pattern with high levels of cytokines, similar to the cytokine storm in human severe infection (Chan et al., 2020).

Neutralizing antibodies induced by primary SARS-CoV-2 infection protected hamsters from subsequent infection. Moreover, passive serum transfer also protected naive hamsters against viral replication in the lung (Imai et al., 2020). SARS-CoV-2 was transmitted efficiently between individual hamsters by direct contact and *via* aerosols (Sia et al., 2020). Therefore, Syrian hamsters can be used for research on viral transmissibility. At present, the replicative abilities, pathogenicity, transmissibility, and susceptibility to neutralization of some virus variants (P.1, D614G, and a variant with a deletion at the S1/S2 junction of the spike protein) have been evaluated in hamsters (Hou et al., 2020; Imai et al., 2021; Plante et al., 2021; Wang et al., 2021).

Moreover, the hamster model appears to mimic the gender bias and age-dependent differences in COVID-19 patients (Osterrieder et al., 2020; Yuan et al., 2021). Younger animals launched earlier and more severe lesions and a faster recovery after infection (Osterrieder et al., 2020). Male hamsters showed relatively severe clinical symptoms and more severe lung damage, while female hamsters behaved like human asymptomatic carriers with lower levels of virus replication. Higher receptor binding domain (RBD)-specific IgG levels in female hamsters indicated that the stronger humoral immune response in female contributes to reducing disease severity (Yuan et al., 2021). Therefore, Syrian hamster is an appropriate animal model for developing gender-based and age-different treatments of COVID-19.

It would be interesting to incorporate into these models known risk factors for severe COVID-19, such as metabolic syndrome, respiratory disease, hypertension, and old age, which might produce valid models of COVID-19 with comorbidities (Williamson et al., 2020). For example, the detrimental impact of a continuous Western diet on COVID-19 outcome has been proved in Syrian hamster (Port et al., 2021).

### Roborovski Dwarf Hamster (*Phodopus roborovskii*)

**FIGURE 1:**
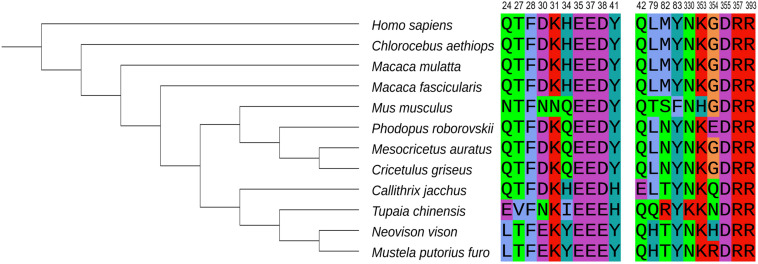
Phylogenetic analysis of ACE2 orthologs of 12 species and crucial ACE2 residues at the interface with the viral S protein. National Center for Biotechnology Information (NCBI) accession numbers for ACE2 are as follows: NP_001358344.1 (human), XP_008987241.1 (marmoset), XP_027288607.1 (Chinese hamster), XP_005593094 (cynomolgus macaque), NP_001129168.1 (rhesus macaque), XP_005074266.1 (Syrian hamster), NP_001123985.1 (mouse), BAE53380.1 (ferret), QPL12211.1 (mink), QPL07045.1 (Roborovski dwarf hamster), XP_006164754.1 (tree shrew), and AAY57872.1 (African green monkey) were analyzed and the phylogenetic tree was accomplished by MGEA-X (version 10.0.5). The 20 amino acid residues at the interface between human ACE2 and SARS-CoV-2 RBD are shown on the right (Shang et al., 2020). Each amino acid is identified by a different color.

Roborovski dwarf hamster, the smallest hamster in the world, has also proven to be a highly susceptible model of SARS-CoV-2. Hamsters infected with 10^5^ plaque forming units (PFU) SARS-CoV-2 developed a rapid onset of fulminant clinical disease, with a sudden fall of body temperatures at 1–2 dpi and severe acute diffuse alveolar damage and hyaline microthrombi in the lung (Table 1; Trimpert et al., 2020). The lungs, jejunal, and kidneys of Roborovski dwarf hamsters contained high virus loads. Interestingly, Trimpert et al. (2020) found that Roborovski dwarf hamsters infected with low doses (5,000 PFU) still developed infection, but hyaline thrombi were not observed in such hamsters. It appears that Roborovski dwarf hamsters are highly susceptible to SARS-CoV-2 even at low doses, and mimic the clinical and pathological outcome observed in severe cases of COVID-19 (Table 2; Trimpert et al., 2020). Because the amino acid sequence at the ACE2-RBD interface of Roborovski dwarf hamster differs from the Syrian hamster by only one amino acid (Figure 1), this minor difference is inadequate to explain its high susceptibility to SARS-CoV-2 and its fatal outcomes. Perhaps it is related to the difference in the host’s immune response. Unfortunately, there is not enough research to clarify this issue due to the lack of dedicated tools and reagents for Roborovski dwarf hamster.

### Chinese Hamster (*Cricetulus griseus*)

Chinese hamsters are smaller than Syrian hamsters, and have been tested for SARS-CoV-2 infection (Bertzbach et al., 2020). Decreases in body weights post infection were significant and infected hamsters failed to regain lost weight by the end of the experiment at 14 dpi. Histopathology revealed pneumonia and alveolar damage similar to what has been observed in Syrian hamsters (Table 1; Bertzbach et al., 2020). However, the course of bronchitis and pneumonia described in Chinese hamsters was milder compared to Syrian hamsters, and pneumonia was more prolonged. Evident acute alveolar damage was present until 14 dpi with persistence of viral RNA (Bertzbach et al., 2020). Chinese hamsters are highly susceptible to diabetes, which may be used to establish COVID-19 model with preexisting diabetes (Gerritsen, 1982).

Owing to the similarities to COVID-19 patients regarding clinical symptoms and histopathology, hamster-based models have become important tools for the study of SARS-CoV-2. Various drugs, antibodies, and vaccines are already being tested in Syrian hamsters and progresses have been reported (Kaptein et al., 2020; Kreye et al., 2020; Rogers et al., 2020; Kim et al., 2021; Ku et al., 2021).

## Mouse

Compared with other lab animals, mice offer many practical advantages including small sizes, multiple well-established strains, clear genetic background, highly available research tools, and ease of genetic manipulation. Due to the low affinity of mouse ACE2 (mACE2) for S protein of SARS-CoV-2, mice cannot be efficiently infected with wild type viruses (Wan et al., 2020). Two main approaches were adopted for circumventing the incompatibility of mACE2 with SARS-CoV-2 S protein: (1) the use of mouse-adapted SARS-CoV-2 strains; (2) expression of human ACE2 (hACE2) in mice.

### Mouse-Adapted SARS-CoV-2 Strains

SARS coronavirus clinical isolates successively passaged in the respiratory tract of mice generated mouse-adapted strains MA15 and v2163, which caused lung damage and death in mice (Roberts et al., 2007; Day et al., 2009). Gu et al. (2020) used the same strategy and produced mouse-adapted SARS-CoV-2 strain MASCp6, which was infectious to mice and caused inflammatory responses and moderate pneumonia in both young and aged mice. The adaptive mutation of the strain, Asn501 to Tyr, at the key site in the RBD of viral S protein increased the binding affinity of the protein to mACE2. Another mouse-adapted strain, HRB26M, only caused mild pathological changes in lung, same as MASCp6 (Wang et al., 2020). This method enables fast establishment of mouse model of infection to provide a large number of experimental animals that can be directly applied to the evaluation of therapeutic and vaccine candidates. However, wild type mice infected with mouse-adaptive virus strains may not necessarily reflect the situation of wild type SARS-CoV-2 infection (Table 2; Daniloski et al., 2020).

Dinnon et al. (2020) used reverse genetics to remodel the S and mACE2 binding interface and constructed a recombinant virus (SARS-CoV-2 MA) that utilizes mACE2 for entry. This recombinant strain replicated in the respiratory tract and was cleared within 4 dpi, causing mild to moderate pneumonia (Table 1). Leist et al. (2020) obtained a new mouse-adapted SARS-CoV-2 virus with increased pathogenicity, SARS-CoV-2 MA10, *via* serial passage of SARS-CoV-2 MA in the lungs of mice. MA10 infection caused acute lung injury and partial mortality in young BALB/c mice. The mortality rate of standard infection dose (10^4^ PFU) was 20% (Leist et al., 2020). Compared to mice with MA infection, more severely impaired lung function, significant changes in PenH (enhanced pause) and Rpef (the fraction of expiration time at which the peak occurs) at 2 dpi, was observed in MA10 infected adult mice. Besides, the levels of several pro-inflammatory cytokines (Table 1) in the lungs of aged adult mice with SARS-CoV-2 MA or MA10 infection, rather than in the serum, increased at 2 dpi. This more localized cytokine/chemokine responses may explain the longer time of virus clearance in aged mice than in young mice (Dinnon et al., 2020; Leist et al., 2020). The above two mouse-adapted strains exhibit an age-dependent disease severity in mice similar to COVID-19 patients (Table 2; Dinnon et al., 2020; Leist et al., 2020). In comparison to BALB/c mice, the infection of MA 10 led to less severe and non-lethal disease in C57BL/6J mice, revealing host genetic background could affect SARS-CoV-2 disease susceptibility (Leist et al., 2020). Recently, a mouse-adapted strain WBP-1 was found to produce lethal lung infections in mice. Q493K and Q498H substitutions in S protein of WBP-1 increased the binding affinity toward mACE2 (Huang et al., 2021).

In order to assess the anti-SARS-CoV-2 efficacy of remdesivir, which inhibits viral RNA-dependent RNA polymerase (RdRp), Pruijssers et al. (2020) constructed a chimeric mouse-adapted SARS-CoV variant, SARS/SARS2-RdRp, by replacing SARS-CoV RdRp with SARS-CoV-2 RdRp. This method is suitable for evaluating drugs with known target.

### Transgenic Mice

Various antiviral agents and vaccines have so far been evaluated in transgenic mouse models for COVID-19 (Liu et al., 2020b; Ma et al., 2020; Colunga Biancatelli et al., 2021). After intranasal inoculation with SARS-CoV-2, transgenic mice expressing hACE2 by the mACE2 promoter showed symptoms of girdling and weight loss, as well as virus replication in the lungs (Bao et al., 2020). Infected mice progressed to moderate interstitial pneumonia with diffuse lesions at 5 dpi, and the pneumonia became mild with focal lesion areas at 7 dpi, while other organs displayed no obvious lesions (Table 1). In addition, a large number of macrophages and CD3^+^ T lymphocytes were found in the alveolar interstitium of hACE2 mice, and their numbers gradually increased with the course of infection (Bao et al., 2020). Another stable mouse model was generated, replacing the endogenous mACE2 with hACE2, by using CRISPR/Cas9 knockin (KI) technology (Sun S.H. et al., 2020). These hACE2 mice are highly susceptible to SARS-CoV-2 and demonstrate more robust virus replication in lung than hACE2 transgenic mice (Bao et al., 2020). Moreover, hACE2-KI mice (C57BL/6 background) with intratracheal inoculation of SARS-CoV-2 developed severe pneumonia with hyaline membranes-like changes, and can be used as an animal model for SARS-CoV-2-induced ARDS (Hong et al., 2021).

Two transgenic mouse models with hACE2 expression driven by the heterologous promoters showed lethal disease (Jiang et al., 2020; Winkler et al., 2020). After intranasal inoculation of the virus, K18-hACE2 mice and HFH4-hACE2 mice (hACE2 expression under the control of human cytokeratin 18 promoter and HFH4/FOXJ1 promoter, respectively) lost weight and generated typical interstitial pneumonia. K18-hACE2 and HFH4-hACE2 mice with SARS-CoV-2 neuroinvasion would succumb to the infection. Severe pneumonia in K18-hACE2 mice was characterized by high levels of pro-inflammatory cytokines and chemokines (IFN-β, IL-6, IL-11, CXCL10, etc.) and substantial inflammatory cell infiltration in the lung. Lung consolidation caused by infection led to reduced exercise tolerance and impaired lung function in those mice (Winkler et al., 2020). HFH4-hACE2 mice with SARS-CoV-2 infection showed increased levels of creatine kinase, which may be related to edema and necrosis in some cardiomyocytes observed in heart tissue of mice (Jiang et al., 2020).

### Viral Vector-Mediated Delivery Systems

Human ACE2 expression in lung tissues of BALB/c mice was also achieved by intranasal administration of replication-defective adenoviruses (AdV) encoding hACE2 (AdV-hACE2). Five days later, mice were inoculated with SARS-CoV-2 and displayed weight loss, high virus replication in the lungs and pulmonary damage (Hassan et al., 2020). Lower levels of SARS-CoV-2 RNA could be detected in brain, liver, and spleen, but not in kidney, gastrointestinal tract, or serum (Table 1). The tissue distribution of virus infection may be related to the expression of hACE2 in different tissues in addition to the natural tropism of the virus (Hassan et al., 2020). Interestingly, Hassan et al. (2020) also found that there was no substantial difference between nasal inoculation alone and intranasal–intravenous combined routes in lung infection. It indicates that virus inoculation by a systemic route is not required in this animal model. Infected mice also showed a substantial increase in neutrophil infiltration in perivascular and alveolar locations after 8 dpi, and mice given anti-IFNARI1 monoclonal antibody showed more severe infection with an increase in macrophage infiltration at 8 dpi, similar to severe pneumonia in COVID-19 patients (Hassan et al., 2020). Those AdV-hACE2 mice treated with anti-Ifnar1 monoclonal antibody had relatively high levels of pro-inflammatory cytokines and chemokines such as IL-6, CCL2, CCL5, CXCL10, CXCL11, IFN-λ, and IFN-β in lung, indicating that inhibiting type I IFN signaling can aggravate inflammation and promote infection.

Sun J. et al. (2020) also used AdV-hACE2 for enabling SARS-CoV-2 infection of BALB/c and C57BL/6 mice, and found that infection in the two strains followed almost the same disease course. Infected BALB/c mice showed ruffled fur, hunching, and difficulty breathing after 2 dpi, and lost up to 20% of body weight at 4–6 dpi, while C57BL/6 mice lost about 10–15%. Histological examination showed that the most severe changes in the lungs were observed at 5 dpi (Sun J. et al., 2020). Compared with AdV-Empty transduced mice, expression of multiple genes were upregulated in lungs of AdV-ACE2 transduced mice infected with SARS-CoV-2, such as several cytokines and chemokines, including TNF, IFN-γ, IL-10, IL-15, IL-6, CCL2, CXCL10, and platelet-derived growth factor subunit B (PDGFb), which was consistent with observations in COVID-19 patients (Table 1; Huang et al., 2020). The effects of several candidate vaccines and antiviral therapeutics (polyinosinic–polycytidylic acid, remdesivir, 1B07 anti-S protein monoclonal antibody, etc.) have been evaluated in this animal model (Alsoussi et al., 2020; Hassan et al., 2020; Sun J. et al., 2020).

Venezuelan equine encephalitis replicon particles (VEEV-VRP) also can be used as an exogenous system for delivering hACE2 (Zhang Y.N. et al., 2020). After SARS-CoV-2 infection, this VEEV-VRP-hACE2 model developed interstitial pneumonia without obvious clinical symptoms, and the virus replication was limited to the lung (Table 2). A human neutralizing antibody CB6 showed strong protective effects against the virus in this model (Zhang Y.N. et al., 2020).

However, delivery of hACE2 with viral vector-mediated delivery systems can cause extra lesions in lung of mice, as mild bronchial inflammation induced by AdV and small areas of alveolar septal thickening induced by VEEV-VRP (Hassan et al., 2020; Zhang Y.N. et al., 2020). It remains a problem that the lung disease caused by viral vector may be confused with the pneumonia caused by SARS-CoV-2. The expression and tissue distribution of hACE2 is not systemic in this kind of models, and may vary among different mice. Consequently, hACE2 mouse model using viral vector-mediated delivery systems is not ideal for simulating systemic SARS-CoV-2 infection (Table 2).

### Others

Severe acute respiratory syndrome coronavirus 2 must be handled in biosafety level three (BSL-3) facilities, which limits the development of urgently needed antiviral agents. Recombinant lentiviral viruses pseudotyped with SARS-CoV-2 S protein as the sole viral envelope protein has been demonstrated to be able to simulate SARS-CoV-2 virus infecting cells through S protein binding to hACE2 (Nie et al., 2020). Fan et al. (2018) reported successful infection of mice transgenic for MERS-CoV receptor using MERS-CoV S pseudovirus. hACE2 transgenic mice with pseudotyped SARS-CoV-2 infection have been applied in COVID-19 research (Cao et al., 2020; Bai et al., 2021). Such pseudoviruses can be handled in BSL-2 facilities and would greatly facilitate studies targeting both SARS-CoV-2 S protein in the context of infection, as well as other SARS-CoV-2 proteins in pathogenesis. Interestingly, recombinant subunit 1 of SARS-CoV-2 S protein (S1SP), rather than the live virus, can induce alveolar and systemic inflammation in K18-hACE2 mice, while the intact S protein provokes minimal or no responses (Colunga Biancatelli et al., 2021). This protein-induced model provides another way to avoid the limitation of BSL-3 facilities for COVID-19.

Hassert et al. (2020) utilized an mRNA-based transfection system for transiently delivering hACE2 into type I interferon receptor 1 deficient (Ifnar1) mice to study the adaptive immune response to SARS CoV-2. hACE2 mRNAs mainly target the lung and liver of mice, and a neutralizing antibody response was detected in those mice after SARS-CoV-2 infection (Hassert et al., 2020). The transfection efficiency of mRNA-ACE2 remains an issue *in vivo* according to the data presented (Hassert et al., 2020).

## Ferret

Ferrets are naturally susceptible to SARS-CoV-2 infection. Infected ferrets showed mild clinical symptoms similar to human, including elevated body temperature, decreased activity, cough, and rhinorrhea (Kim et al., 2020; Park et al., 2020; Shi et al., 2020).

The virus replicated efficiently in the upper respiratory tract of ferrets for up to 8 days (Shi et al., 2020). Viral RNA shedding was detectable up to 18 dpi in high-dose group (5 × 10^6^ PFU) (Ryan et al., 2021). RNA could be detected in ferret’s nasal wash, blood, nasal washes, urine, and fecal specimens, indicating diverse routes of shedding (Kim et al., 2020). Immunohistochemistry detected viral antigens in nasal turbinate, trachea, lung, and intestine (Kim et al., 2020), while histology analysis revealed severe lymphoplasmacytic perivasculitis, vasculitis, and mild peribronchitis in the lung (Table 1; Shi et al., 2020). Compared with pathological changes in COVID-19 patients with pulmonary edema and diffuse alveolar damage, virus-induced damage to ferret lungs is milder (Xu Z. et al., 2020). The mild lung pathology in the high-dose group ferrets was similar to the medium-dose group, but more extensive. No obvious lesions were observed in other tissue, except for multifocal inflammatory cell infiltration was observed in the portal areas of liver (Ryan et al., 2021). Ferrets are fully protected from acute lung pathology following re-challenge of the virus, which indicates naturally acquired immunity helps ferrets against reinfection (Patel et al., 2021; Ryan et al., 2021).

After direct contact with infected ferrets, all healthy naive ferrets showed moderate increases in body temperature and decreases in activity. Naive ferrets in indirect contact with infected ferrets through permeable partition did not show obvious symptoms, but some became positive for viral RNA in nasal washes and fecal specimens. It suggests that SARS-CoV-2 can be transmitted between ferrets through direct contact and airborne route, similar to transmission between humans (Kim et al., 2020; Richard et al., 2020). These results indicate that the ferret model is excellent for studying SARS-CoV-2 transmission and mild infection (Table 2).

## Mink

Severe acute respiratory syndrome coronavirus 2 outbreaks occurred in mink (*Neovison vison*) farms in the Netherlands, with infected minks showing signs of respiratory disease, including watery nasal discharge and even severe respiratory distress ending in death (Oreshkova et al., 2020). The virus was suspected to be transmitted from infected farm workers and then spread among the minks (Oreshkova et al., 2020). Highly diverse mink SARS-CoV-2 variants, including Y453F, F486L, and N501T, are found and spill over to human (van Dorp et al., 2020; Oude Munnink et al., 2021). Escaped and wild minks infected with SARS-CoV-2 might become a virus reservoir, potentially constituting a pandemic threat (Koopmans, 2021).

After displaying clinical symptoms for 1 or 2 days, infected minks usually stopped eating and died on the next day (Molenaar et al., 2020). Histological examination revealed severe diffuse interstitial pneumonia with diffuse alveolar damage and formation of hyaline membranes in the lung (Molenaar et al., 2020; Oreshkova et al., 2020). Viral RNA could be detected in the conchae, lungs, liver, distal large intestine, throat swabs, and rectal swabs (Oreshkova et al., 2020) and SARS-CoV-2 isolated from mink (MT396266) was highly similar to circulating human isolates (Table 1; Sharun et al., 2020). Minks have transmitted SARS-CoV-2 to humans, and their high susceptibility to the virus suggest that they can easily become a natural reservoir (Enserink, 2020).

In laboratory settings, a SARS-CoV-2 strain isolated from human was used to infect minks intranasally (Shuai et al., 2020). Infection caused severe pathological injury similar to COVID-19 in the respiratory tracts of minks, as autopsy revealed severe interstitial pneumonia with extensive and diffuse consolidation, pulmonary thrombosis, nasal mucosa damage in the olfactory region, and nasal cavities filled with mucinous-purulent secretion. Immunohistochemical analysis of lung lobes showed the widespread distribution of SARS-CoV-2 antigen-positive cells; and high levels of virus replication were detected in turbinates, soft palate, lung lobes, and other tissues (Table 1; Shuai et al., 2020). The virus can be transmitted between minks *via* the air over more than 1 m distance (Kutter et al., 2021). Although minks infected in the laboratory did not die like infected farm minks, they lost 10–20% of body weight at around 8 dpi. In addition, a spike protein-based subunit vaccine candidate was evaluated using this mink model (Shuai et al., 2020).

## Non-Human Primates

### Rhesus Macaque (*Macaca mulatta*)

After inoculation with SARS-CoV-2 *via* a combination of intratracheal, intranasal, ocular, and oral routes, rhesus macaques showed changes in respiratory pattern (irregular, accelerated respiration), piloerection, and weight loss on 1 dpi (Table 1; Munster et al., 2020a). Pulmonary infiltrates were visible in lung radiographs of all animals, similar to what have been observed in patients with COVID-19. The symptoms could last 8–16 days and autopsy revealed pulmonary edema, pulmonary thrombosis, and interstitial pneumonia centered on terminal bronchioles (Munster et al., 2020a; Shan et al., 2020). Some rhesus monkeys generated neutralizing antibodies after infection, providing protection from secondary infection, which proved that humoral immunity produce a marked effect (Shan et al., 2020). Cytokine and chemokine levels in serum, such as IL-1ra, IL-6, IL-10, IL-15, MCP-1, macrophage inflammatory protein (MIP)-1β increased on 1 dpi, but minor decline was observed in transforming growth factor (TGF)-α on 3 dpi (Munster et al., 2020a). Virus loads were high in nasal swabs, throat swabs, and bronchoalveolar lavage fluids. Urogenital swabs remained negative in all animals, and only one rhesus monkey showed virus shedding from the rectum on 21 dpi. These observations indicate that the rhesus macaque infection model replicates moderate symptoms of COVID-19 patients. On the other hand, old macaques showed more severe diffuse interstitial pneumonia compared to young macaques and may thus be used for the study of more severe SARS-CoV-2 infection (Table 2; Yu et al., 2020).

### Cynomolgus Macaque (*Macaca fascicularis*)

In one study, cynomolgus macaques inoculated with SARS-CoV-2 displayed increased body temperature and weight loss (Lu et al., 2020). In another similar study, however, the inoculated animals showed no overt clinical symptoms, except for a serous nasal discharge in one aged animal on 14 dpi (Rockx et al., 2020). This difference may be related to different methods and doses of virus inoculation. Both studies reported diffuse interstitial pneumonia. Besides, Lu et al. (2020) also found inflammation in liver and heart, and mild lesions in mesenteric lymph nodes and kidney (Table 1). By 14 dpi, there were virus-specific antibodies against the S and nucleocapsid proteins in the serum of all animals (Rockx et al., 2020). Viral shedding in upper respiratory tract of aged animals was prolonged to 21 dpi (Rockx et al., 2020). Compared with young adult animals, higher levels of viral RNA were detected in nasal swabs of aged animals. However, aged animals did not show more prominent lung infections or obvious clinical symptoms, which was different from rhesus macaques (Lu et al., 2020; Rockx et al., 2020).

### African Green Monkey (*Chlorocebus aethiops*)

African green monkeys (AGMs) supported a higher level of viral replication than rhesus and cynomolgus macaques, for both SARS-CoV and SARS-CoV-2 (McAuliffe et al., 2004; Woolsey et al., 2021). After virus challenge, AGMs showed mild and various clinical symptoms, including decreased appetite, elevated body temperature, and even severe respiratory distress and death in two aged animals. Lung tissues developed extensive interstitial pneumonia with hyperemia and hemorrhage, and more severe lesions with ARDS was observed in two of four aged animals (Blair et al., 2021; Woolsey et al., 2021). Aged AGMs may become a lethal model to mimic serious COVID-19 manifestations, including ARDS. Mild liver inflammation and chronic bilateral glomerulonephritis was noted in extrapulmonary organs of infected AGMs. The changes of systemic cytokines in AGMs following infection is consistent with COVID-19 patients: IFN-stimulated genes and IL-6 and IL-8 signaling upregulated (Woolsey et al., 2021). A study have evaluated the transmissibility and disease severity of SARS-CoV-2 B.1.1.7 variant in AGMs (Rosenke et al., 2021).

### Common Marmoset (*Callithrix jacchus*)

Common marmoset, a New World monkey, has been used in research on a wide range of human diseases (Zühlke and Weinbauer, 2003; Adams et al., 2008). Compared with other non-human primates, it has smaller size, breeds well in captivity, and can be handled with ease. SARS-CoV-2 inoculated marmoset displayed no obvious clinical symptoms, with only one-third of animals showing slightly elevated body temperature (Table 2; Lu et al., 2020). Viral RNA could be detected in the peripheral blood from 2 to 8 dpi, and in nasal, throat, and anal swabs during 2 weeks post viral inoculation. Slight infiltration of inflammatory cells into pulmonary septum and mild lesions in liver and spleen were observed in histopathological examination (Table 1; Lu et al., 2020). Although no viral RNA was detected in tissue samples from the two tested marmosets, ultrastructural lesions in the lung and heart were observable under transmission electronic microscope (Lu et al., 2020). Additionally, virus-specific antibody was undetectable in the serum of marmosets during the experiment. These results indicate that, in contrast to cynomolgus and rhesus macaques, common marmoset is relatively resistant to SARS-CoV-2 infection and may not be a suitable animal model.

## Comparison of Animal Models

### Susceptibility and Disease Severity

ACE2 is a crucial factor that limits the sensitivity of the host to SARS-CoV-2. We select 11 species currently being used as SARS-CoV-2 animal models, and analyze the amino acid sequence of their ACE2 to construct a phylogenetic tree (Figure 1). The amino acid residues shown in Figure 1 can form hydrogen bonds or salt bridges with S protein to stabilize the binding (Shang et al., 2020; Liu et al., 2021). In primates, the 20 amino acid residues at the interface of human and the Old World monkeys, are exactly the same. But marmoset, a New World monkey, have lower residue conservation, which may explain its relatively resistance to SARS-CoV-2 infection. In addition, tree shrew are less susceptible to the virus (Liu et al., 2021).

The difference in ACE2 can partially explain the susceptibility of some species to viruses. Liu et al. (2021) found an interesting phenomenon: ACE2 of ferret and mink, which exhibit limited binding affinity with viral S1 protein, have the function of mediating the entry of SARS-CoV-2 in cell-level analysis. However, farm minks developed fatal infections, which constitutes a model of severe and potentially lethal infection of SARS-CoV-2 (Oreshkova et al., 2020). Therefore, the actual host–pathogen interaction is more complicated.

In summary, among the several animal models, three types of hamsters, ferret, mink, AGMs, rhesus, and cynomolgus macaque are highly susceptible to SARS-CoV-2, while marmoset, tree shrew, and wild-type mouse are relatively less susceptible.

Susceptible small animal models for COVID-19 are of great significance in evaluating antiviral agents and vaccines. Syrian hamster infected with high-dose virus and *RAG2* KO hamsters developed lethal disease (Brocato et al., 2020; Tostanoski et al., 2020). Although wild-type mouse cannot be infected with SARS-CoV-2 in its natural state. Some mouse models (BALB/c mice infected with MA10, K18-hACE2, and HFH4-hACE2 mice) so far have reproduced severe respiratory disease that resemble pneumonia in severe COVID-19 in humans. In view of the time required for the development and production of transgenic mice, which usually takes from several months to years, approaches based on mouse-adapted or recombinant virus strains capable of infecting through mACE2, or expression of hACE2 in mouse lung cells are generally much quicker, and susceptible animals can be obtained in as fast as 2–3 weeks (Sun J. et al., 2020).

### Lung Pathology

Typical pathological changes in the lung of COVID-19 patients include pulmonary edema with hyaline membrane formation, diffuse alveolar damage, type II pneumocyte hyperplasia, dilatation of capillary and occurrence of thrombosis, monocytes and macrophages within alveolar spaces, and interstitial mononuclear inflammatory infiltrates. Pulmonary fibrosis and consolidation in COVID-19 patients are usually milder compared to SARS patients, but mucus secretion tends to be more prominent in the former (Zhou B. et al., 2020). The most common patterns of COVID-19 pneumonia on chest CT were ground-glass opacity and bilateral patchy shadowing (Xu X. et al., 2020).

Mild pulmonary abnormality of tree shrew failed to recapitulates the typical characteristics of COVID-19. On the other hand, hamsters are promising models in this aspect. Syrian hamster infected with SARS-CoV-2 showed moderate to lethal broncho-interstitial pneumonia and bilateral ground-glass opacity on CT (Table 1), similar to the pneumonia characteristics of COVID-19 (Imai et al., 2020; Tostanoski et al., 2020). The lung pathology of Chinese hamster was roughly similar to that of Syrian hamster, but the pneumonia was milder and prolonged (Bertzbach et al., 2020). Notably, typical manifestations of ARDS, including severe acute diffuse alveolar damage and hyaline microthrombi, have been observed in infected Roborovski dwarf hamsters, providing the opportunity for studying SARS-CoV-2-related ARDS (Trimpert et al., 2020). Mild to severe interstitial pneumonia has been observed in different mouse models (Table 1). In contrast to K18-hACE2 transgenic mice, AdV-hACE2 mice develop lower titer viral replication and less obvious symptoms. Destroying the intact type I IFN response exacerbated lung pathology and clinical symptoms of AdV-hACE2 mice (Hassan et al., 2020; Rathnasinghe et al., 2020). Minks and ferrets, the closely related mustelids, displayed different patterns of viral replication (Shuai et al., 2020). SARS-CoV-2 replicated efficiently in both the upper and lower respiratory tract of minks, but only in the upper respiratory tract of ferrets (Shi et al., 2020; Shuai et al., 2020). Consequently, acute bronchiolitis in infected ferrets, is less similar to that of COVID-19 patients. Among all animals that have been used for modeling SARS-CoV-2 infection, three kinds of Old World monkeys are closest to human in terms of lung pathology, and common marmoset shows relatively milder lung damage.

### Extrapulmonary Pathology

Some COVID-19 patients, especially those with severe infection, have extrapulmonary symptoms, such as kidney injury, cardiac complications, and neurologic manifestations (dizziness, impaired consciousness, taste impairment, smell impairment, etc.) (Chen N. et al., 2020; Mao et al., 2020; Zhou F. et al., 2020). Although the main target organ of SARS-CoV-2 is lung, the virus has a broad organotropism and can be detected in some extrapulmonary organs, including the kidneys, liver, heart, and brain, according to the result of autopsy (Puelles et al., 2020). Pathology results showed a few interstitial mononuclear inflammatory infiltrates in heart tissue and severe acute tubular necrosis in kidney tissue (Zhou B. et al., 2020). In addition, lymphocytic panencephalitis, meningitis, ischemic damage, and microinfarcts were reported in postmortem brain of COVID-19 patients (von Weyhern et al., 2020; Song et al., 2021).

Although some animal models develop extrapulmonary manifestations of disease after infection as previously mentioned (Table 1), there is still a lack of an ideal model that can mimic the systemic response to SARS-CoV-2 infection in human.

Acute tubular necrosis, mild focal myocardial degeneration, and adrenal medulla atrophy could be observed in extrapulmonary tissues of Syrian hamsters (Song et al., 2020). Syrian hamster has been used in the study of myocardial pathology in COVID-19 (Chen S. et al., 2020). SARS-CoV-2 infection of cardiomyocytes, increased CCL2 expression and macrophage infiltration were reported in their heart tissues, which was confirmed in autopsy samples of COVID-19 patients. This study provides direct evidence that SARS-CoV-2 infects cardiomyocytes *in vivo*. Therefore, Syrian hamsters may be applied in pathological research of systemic SARS-CoV-2 infection.

In addition, SARS-CoV-2 is proved neurotropic in some mouse models. After intranasal inoculation of the virus, 6-week-old K18-hACE2 mice showed some neurological symptoms, such as tremors and ataxic gait, and all animals died by 6 dpi (Kumari et al., 2021). Those mice also exhibited encephalitis features including production of cytokines and chemokines, leukocyte infiltration, and neuronal cell death (Kumari et al., 2021). SARS-CoV-2 could infect cells within the nasal turbinate, eye, and olfactory bulb, which related to smell impairment induced by infection of these cells in COVID-19 patients (Kumari et al., 2021). Besides, Song et al. (2021) observed the remodeling of brain vasculature in infected regions in this model. In conclusion, the severe neurological disease observed in K18-hACE2 mice is closely linked with the infection of nervous system in human patients, and this mouse model provide a valuable tool for studying the pathogenesis of infection in nervous system. The primary manifestation of clinical disease in these hACE2 transgenic mouse models was encephalitis, rather than that severe pneumonia has been reported in some hamster model, which limits them to systemic SARS-CoV-2 models (Tostanoski et al., 2020). Neuroinvasion also could be found in mice which expressed adeno-associated virus (AAV)-hACE2 expression in brain and then infected with SARS-CoV-2 intraventricularly. Those mice developed weight loss and death after infection (Song et al., 2021).

### Host Immune Cellular Profile

The counts of white blood cells in the peripheral blood of COVID-19 patients are normal or decreased in the early stage of onset with the decrease in lymphocytes, and the progressive decrease in lymphocytes and increased inflammatory factors can be observed in severe patients (Huang et al., 2020).

According to the whole genome sequencing, the immune and nervous systems of tree shrew have high homology with those of human (Fan et al., 2013). In addition, some cytokines of tree shrew are similar to their human counterparts in terms of structure and function, such as IFN-λ3 and CXCL12 (Li et al., 2013; Chen et al., 2014). However, instead of lymphopenia often observed in human patients, tree shrews in the adult group increased white blood cells, lymphocytes, monocytes, and granulocytes but old group had a significant decrease in monocytes (Xu L. et al., 2020). The overall pattern of immune cellular profile is different from that in humans.

Shan et al. (2020) noticed leukocytes and neutrophils decreased, but lymphocytes increased in the blood of infected rhesus macaques. In another experiment, leukocytosis, neutrophilia, monocytosis, and lymphopenia were observed at 1 dpi, and then lymphocytes and monocytes returned to baseline, but the neutrophils continued to decline by 5 dpi (Munster et al., 2020a). In conclusion, rhesus macaques infected with SARS-CoV-2 showed neutropenia, lymphocytopenia, and increased NLR, similar to that observed in severe COVID-19 patients (Cao, 2020). And the number of T cells and monocytes in cynomolgus macaques peaked at 2 or 4 dpi, then gradually decreased and reached the lowest at 10 or 12 dpi, consistent with transient lymphocytopenia in rhesus macaques (Lu et al., 2020).

## Conclusion

A variety of animal models for COVID-19 have been developed. Most animal models have mild to moderate infections. Minks, AGMs, certain mouse, and hamster models with critical infections can be applied to the pathogenesis study of severe COVID-19. Animals with low ACE2-binding affinity, such as mice, can be made susceptible to SARS-CoV-2 through engineering with expression of human hACE2 (transgenic technology or viral vector-mediated delivery systems) or by using adapted/recombinant virus strains.

The age-dependent increase in disease can be observed in some animal models. Researchers can consider age and other additional risk factors of COVID-19 to be incorporated into models to mimic human comorbidities. In addition, animal models administered with pseudovirus or viral proteins (S1SP) which are not restricted by BSL-3 facilities can be established and used for research on COVID-19 pathogenesis.

## Author Contributions

SS and YX conceptualized the review. SS drafted the manuscript. YX, SS, ML, YY, NK, YS, DT, NL, FW, and JL revised the manuscript critically for important intellectual content and approved the version of the manuscript to be published. All authors contributed to the article and approved the submitted version.

## Conflict of Interest

The authors declare that the research was conducted in the absence of any commercial or financial relationships that could be construed as a potential conflict of interest.

## Publisher’s Note

All claims expressed in this article are solely those of the authors and do not necessarily represent those of their affiliated organizations, or those of the publisher, the editors and the reviewers. Any product that may be evaluated in this article, or claim that may be made by its manufacturer, is not guaranteed or endorsed by the publisher.
